# Supplementary material for: Mentha Rhizomes as an Alternative Source of Natural Antioxidants
Source: Molecules. 2020 Jan 3;25(1):200. doi: 10.3390/molecules25010200 (PMC6983171; doi:10.3390/molecules25010200)
Supplement: Supplementary file 1 [file molecules-25-00200-s001.pdf]

Supplementary material

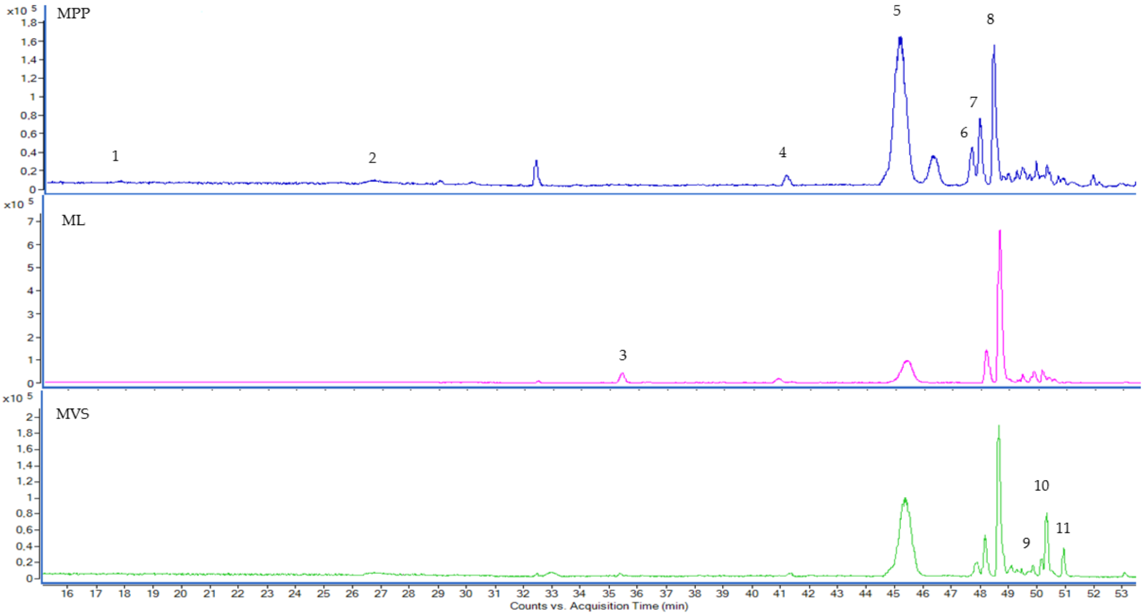

Figure S1 Total ion current (TIC) chromatogram of rhizomes infusions of three *Mentha* L. species: *Mentha* × *piperita* cv. 'Perpeta' (MPP), *Mentha longifolia* (ML) and *Mentha* × *villosa* cv. 'Snežná' (MVS) from the ESI-MS in negative mode: protocatechuic aldehyde (1), caffeic acid (2), eriodictyol-7-*O*-rutinoside (3), 2-(3,4-dihydroxyphenyl)ethyl ester of Salvianolic acid D (4), rosmarinic acid (5), hesperetin-7-*O*-rutinoside (6), salvianolic acid B (7), lithospermic acid (8), salvianolic acid A (9), caffeic acid tetramer (10), luteolin-7-*O*-rutinoside (11).
